# Comparative Transcriptomic Analysis and Candidate Gene Identification for Wild Rice (GZW) and Cultivated Rice (R998) Under Low-Temperature Stress

**DOI:** 10.3390/ijms252413380

**Published:** 2024-12-13

**Authors:** Yongmei Yu, Dilin Liu, Feng Wang, Le Kong, Yanhui Lin, Leiqing Chen, Wenjing Jiang, Xueru Hou, Yanxia Xiao, Gongzhen Fu, Wuge Liu, Xing Huo

**Affiliations:** 1 College of Agriculture, South China Agricultural University, Guangzhou 510642, China; yuyongmei111@163.com (Y.Y.); 15112088151@163.com (W.J.); xyx13759419730@163.com (Y.X.); 13617766417@163.com (G.F.); 2 Rice Research Institute, Guangdong Academy of Agricultural Sciences/South China High-Quality Rice Breeding Laboratory (Jointly Established by Ministry of Agriculture and Rural Affairs and Provincial Government)/Guangdong Key Laboratory of Rice Science and Technology/Guangdong Rice Engineering Laboratory, Guangzhou 510640, China; dilin_liu@163.com (D.L.); fwangl631@163.com (F.W.); kongldk@163.com (L.K.); chenleiq0816@163.com (L.C.); hxr118123@163.com (X.H.); 3 Institute of Food Crops, Hainan Academy of Agricultural Sciences, Haikou 571100, China; lyh_1012@163.com

**Keywords:** rice, photosynthesis, starch and sucrose metabolism, coexpression, TFs

## Abstract

Rice is a short-day thermophilic crop that originated from the low latitudes of the tropics and subtropics; it requires high temperatures for growth but is sensitive to low temperatures. Therefore, it is highly important to explore and analyze the molecular mechanism of cold tolerance in rice to expand rice planting areas. Here, we report a phenotypic evaluation based on low-temperature stress in indica rice (R998) and wild rice (GZW) and a comparative transcriptomic study conducted at six time points. After 7 days of low-temperature treatment at 10 °C, R998 exhibited obvious yellowing and greening of the leaves, while GZW exhibited high low-temperature resistance, and the leaves maintained their normal morphology and exhibited no yellowing; GZW has a higher survival rate. Principal component analysis (PCA) and cluster analysis of the RNA-seq data revealed that the difference in low-temperature resistance between the two cultivars was caused mainly by the difference in low-temperature treatment after 6 h. Differential expression analysis revealed 2615 unique differentially expressed genes (DEGs) in the R998 material, 1578 unique DEGs in the GZW material, 1874 unique DEGs between R998 and GZW, and 2699 DEGs that were differentially expressed not only between cultivars but also at different time points in the same material under low-temperature treatment. A total of 15,712 DEGs were detected and were significantly enriched in the phenylalanine metabolism, photosynthesis, plant hormone signal transduction, and starch and sucrose metabolism pathways. These 15,712 DEGs included 1937 genes encoding transcription factors (TFs), of which 10 have been identified with functional validation in previous studies. In addition, a gene regulatory network was constructed via weighted gene correlation network analysis (WGCNA), and 12 key genes related to low-temperature tolerance in rice were identified, including five genes encoding TFs, one of which was identified and verified in previous studies. These results provide a theoretical basis for an in-depth understanding of the molecular mechanism of low-temperature tolerance in rice and provide new genetic resources for the study of low-temperature tolerance in rice.

## 1. Introduction

Rice is the main food crop in several countries worldwide, and China is the world’s largest rice producer [1]. Rice is a short-day thermophilic crop that originated from the low latitudes of the tropics and subtropics; it requires high temperatures for growth and is sensitive to low temperatures [2]. The most suitable temperature range for rice growth is 25–30 °C. When the ambient temperature is lower than 15 °C, rice plants are subjected to chilling injury, which leads to stagnation of rice growth, fertility disorders, and, in severe cases, rice plant death [3]. The increase in cold tolerance can promote the expansion of rice to colder areas and reduce pressure on crops under traditional climatic conditions; thus, low-temperature chilling injury is a major problem in rice cultivation in many countries [4]. With global climate change and the increasing frequency of extreme weather, the impact of low-temperature stress on rice production is expected to increase. The perception of cold stress begins with protein receptors on the plasma membrane [5]. Cold stress can lead to a decrease in cell membrane fluidity, which leads to changes in membrane protein conformation, the accumulation of metabolites, and changes in the intracellular redox state. Changes in cell membrane fluidity and membrane protein conformation are considered the first physical changes in plants at low temperatures. Low temperatures change the membrane phase of the cell membrane, inhibit the normal operation of cell function, and subsequently affect the growth and development of plants [6]. When plants suffer from cold injury, the content of reactive oxygen species (ROS) in the body increases, which results in damage to plants [7]. In the process of long-term exposure to adverse factors such as chilling injury, plants form a complex system to regulate the ROS content in the body, and superoxide dismutase (SOD) is an indispensable part of this system [8]. Research on improving the low-temperature tolerance of different green plants revealed that those with strong low-temperature tolerance generally presented relatively high SOD activity. Notably, the regulatory effects in the plant body are limited and cannot indefinitely alleviate the damage to plants caused by low temperatures.

RNA-seq, also known as transcriptome sequencing, is a method for sequencing the total mRNA in a cell or tissue, reflecting the genes expressed at different life stages, in different tissue types, in different physiological states, and under different environmental conditions [9]. With the continuous development of sequencing technology, transcriptome sequencing methods have been widely used in the study of cold tolerance in rice. For example, through RNA-seq analysis of overexpressing *DaCBF4* and *DaCBF7* rice lines, a total of 24 cold tolerance genes were identified [10]. RNA-seq technology has revealed that genes related to fiber synthesis and lipid metabolism are expressed in many cold-tolerant cultivars and that cold-tolerant cultivars present increased calcium ion signaling, photosynthetic efficiency, and antioxidant capacity [11]. Zhang et al. used cold-tolerant chromosome fragment substitution lines (CSSLs) derived from common wild rice in Guangxi, China, to analyze the RNA-seq of the cold-tolerant strain DC90 and the sensitive parent 9311 after cold stress at the seedling stage [12]. The results revealed that a total of 659 DEGs were identified in the two lines, and Kyoto Encyclopedia of Genes and Genomes (KEGG) metabolic pathway enrichment analysis showed that these differentially expressed genes (DEGs) were enriched mainly in the phenylpropanoid biosynthesis, ribosomal translation, plant hormone signaling, and photosynthesis pathways. These genes synergistically participate in the cold response mechanism at the seedling stage of rice. When the Oro (cold-tolerant) and Tio Taka (cold-sensitive) rice varieties were used as experimental cultivars for transcriptome sequencing after cold treatment at the seedling stage, a total of 259 and 5579 cold-responsive DEGs were found in Oro and Tio Taka, respectively. GO enrichment analysis showed that 27% of these DEGs were enriched in metabolic processes, 21% in cellular processes, 30% in binding activities, and 22% in catalytic activities; 14 cold response-specific genes were identified [13]. *Oryza sativa* ssp. japonica cv and indica rice 9311 DEGs were analyzed via transcriptome sequencing under low-temperature stress, and significant enrichment of “stimulus-response” substances in the biological process category was observed, suggesting that the japonica rice cultivar *O. sativa* ssp. japonica cv induced a stronger stress response at the transcriptional level [14].

Rice is a light- and temperature-loving plant that is sensitive to temperature throughout its growth and development period, from the seed germination stage to the mature harvest stage, exhibiting susceptibility to low-temperature injury [15]. During spring sowing, “inverted spring cold” leads to a significant reduction in the rice seed germination rate and seedling survival rate, resulting in a lack of seedlings and dead seedlings in a large area of rice, which has a very large effect on the yield and quality of rice [16]. With the intensification of global climate change, low-temperature disasters are occurring more frequently. Therefore, low-temperature stress has become one of the main factors limiting rice production in major rice-producing areas worldwide. Although RNA-seq has been widely used in functional genomic research on cold tolerance in rice, these studies have focused mainly on cultivated rice, and few studies have focused on wild rice. In this study, with indica rice (R998) and wild rice (GZW) used as research cultivars, phenotypic, physiological, and biochemical indices were determined, and RNA-seq was carried out via low-temperature treatment at the seedling stage. Principal component analysis (PCA), cluster analysis, differential expression analysis, enrichment analysis, and transcription factor (TF) analysis were performed on the sequencing data; the core regulatory network was identified via weighted gene correlation network analysis (WGCNA) and qRT–PCR; and key candidate genes were identified. These results provide a theoretical basis for an in-depth understanding of the molecular mechanism of low-temperature tolerance in rice and provide new genetic resources for the study of low-temperature tolerance in rice.

## 2. Results

### 2.1. Phenotypic and Physiological Indices of R998 and GZW Were Determined at Low Temperature

**Figure 1:**
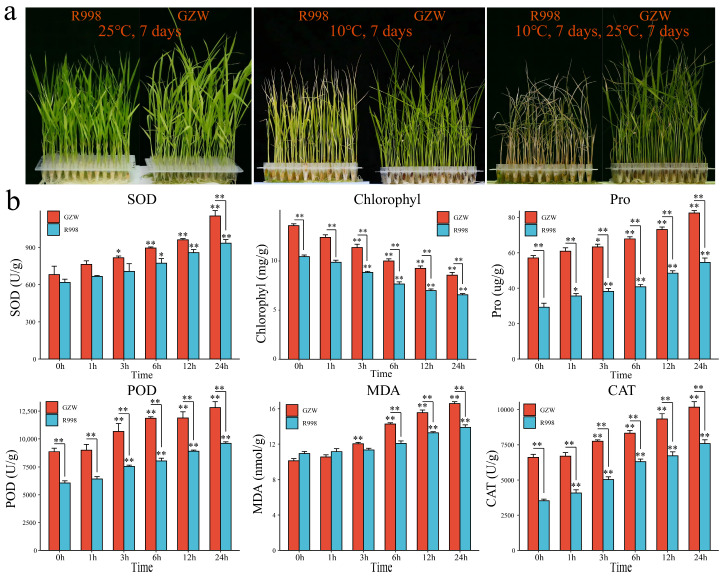
Phenotypic and physiological indices for cold tolerance in R998 and GZW rice. (**a**) Phenotypes of rice treated at 25 °C or 10 °C for 7 days followed by 25 °C for an additional 7 days. (**b**) Changes in the physiological indices of R998 and GZW under low-temperature stress; the results are presented as the means ± SDs (n = 3, * *p* < 0.05, ** *p* < 0.01).

When the air temperature was 25 °C, there was no obvious yellowing observed in the R998 and GZW leaves (Figure 1a). When the air temperature was maintained at 10 °C for 7 days, R998 presented yellowing and loss of green pigmentation in leaves, whereas GZW leaves maintained a normal morphology without any obvious chilling injury symptoms (Figure 1a). After 7 days of treatment at 10 °C and 7 days at 25 °C, R998 not only presented severe leaf margin curling and drying but also obvious yellowing, whereas GZW presented leaf margin drying but not yellowing and could grow normally. The survival rate of GZW was significantly greater than that of R998 (Figure S1). We found that the activities of superoxide dismutase (SOD), proline (Pro), peroxidase (POD), malondialdehyde (MDA), and catalase (CAT) in rice increased significantly with increasing treatment duration under 10 °C stress, and the activities in GZW were significantly greater than those in R998 (Figure 1b). The chlorophyll content decreased significantly and was significantly greater in R998 than in GZW. On the basis of these results, we found that GZW had greater cold tolerance than R998. To further identify the underlying molecular mechanisms and candidate genes, RNA-seq was performed on GZW and R998 at six time points (0 h, 1 h, 3 h, 6 h, 12 h, and 24 h) under cryogenic stress.

### 2.2. RNA-Seq Analysis and Differential Expression Within Materials

**Figure 2:**
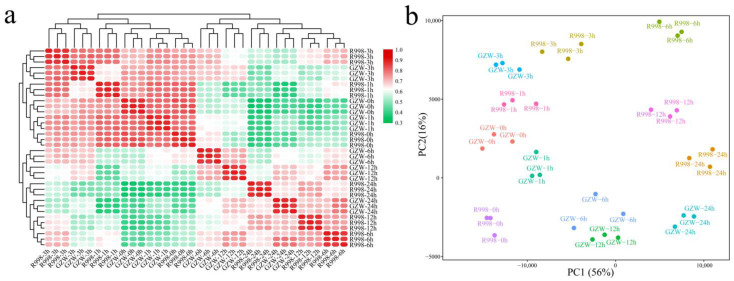
Correlation analysis and PCA of 26 RNA-seq samples. (**a**) Correlation cluster analysis of 36 RNA-seq samples. (**b**) PCA of 36 RNA-seq samples.

A total of 36 samples of the two cultivars subjected to the six durations of low-temperature stress yielded 249.78 Gb of raw data, and after filtering, a total of 304.61 Gb was obtained, and the average Q30 was greater than 96.09% (Table S2). First, correlation analysis of samples was carried out, and the correlations among the three replicates were greater than 0.96 at different temperatures for each material, indicating that the correlation among replicates was high (Figure 2a). By PCA, three replicates of the same sample were combined, and the difference between treatments was greater than the difference between cultivars (Figure 2b). In summary, the correlation between replicates was high, and the results for the different periods were clustered, indicating that the RNA-seq data were reliable and suitable for further analysis.

**Figure 3:**
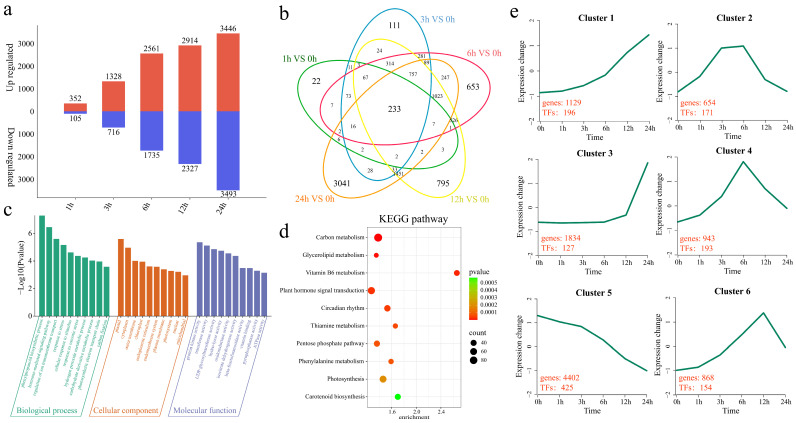
Differential expression and enrichment analysis of GZW. (**a**) Number of genes whose expression was upregulated or downregulated at different time points under low-temperature stress in GZW. (**b**) Venn diagram of the number of common and specific DEGs at different time points of low-temperature stress in GZW. (**c**) GO enrichment analysis of all DEGs at different time points under low-temperature stress in GZW. (**d**) KEGG enrichment analysis of all DEGs at different time points under low-temperature stress in GZW. (**e**) Line graph of the cluster analysis of all DEGs at different time points under low-temperature stress in GZW. The red numbers represent the numbers of DEGs and TFs in each cluster.

First, differential expression analysis of GZW at different time points under low-temperature stress was carried out, and 457 DEGs were identified at 1 h compared with 0 h, of which 352 were upregulated and 105 were downregulated, including 22 unique DEGs (Figure 3a,b). A total of 2044 DEGs were identified at 3 h, of which 1328 were upregulated and 716 were downregulated, including 111 unique DEGs. A total of 4296 DEGs were identified at 6 h, of which 2561 were upregulated and 1735 were downregulated, including 653 unique DEGs. A total of 5241 DEGs were identified at 12 h, of which 2914 were upregulated and 2327 were downregulated, including 795 unique DEGs. A total of 6939 DEGs were identified at 24 h, of which 3446 were upregulated and 3493 were downregulated, including 3041 unique DEGs. To elucidate the functions of the 9830 DEGs in GZW, we analyzed the enrichment of GO terms and KEGG pathways. The significantly enriched GO terms were phenylpropanoid biosynthetic process, hormone-mediated signaling pathway, regulation of ion transmembrane transport, response to stress, cellular response to stimulus, response to osmotic stress, hydrogen peroxide metabolic process, carbohydrate derivative metabolic process, and photosynthetic electron transport chain biological process (Figure 3c). The significantly enriched KEGG pathways included carbon metabolism, circadian rhythm, glycerolipid metabolism, thiamine metabolism, the pentose phosphate pathway, vitamin B6 metabolism, photosynthesis, plant hormone signal transduction, carotenoid biosynthesis, and the phenylalanine metabolism pathway (Figure 3d). The functional changes in genes under low-temperature stress in GZW at different time points were investigated by clustering the 9830 DEGs into six clusters using the k-means clustering algorithm, and the number of TFs in each cluster was determined (Figure 3e). Cluster 1 presented the highest expression level at 24 h, with 196 TFs among the 1129 DEGs. Cluster 2 presented the highest expression level at 3 h and 6 h, with 171 TFs among the 654 DEGs. Cluster 3 presented the highest expression level at 24 h, with 127 TFs among the 1834 DEGs. Cluster 4 presented the highest expression level at 6 h, with 193 TFs among the 943 DEGs. Cluster 5 presented the lowest expression level at 0 h, with 425 TFs among the 4402 DEGs. Cluster 6 presented the lowest expression level at 12 h, with 154 TFs among the 868 DEGs.

**Figure 4:**
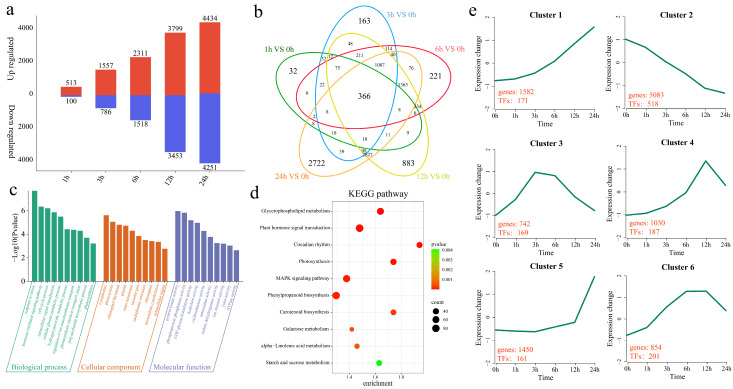
Differential expression and enrichment analysis of R998. (**a**) The number of genes whose expression increased or decreased at different time points under low-temperature stress in R998. (**b**) Venn diagram of the numbers of common and specific DEGs at different time points under low-temperature stress in R998. (**c**) GO enrichment analysis of all DEGs at different time points of low-temperature stress in R998. (**d**) KEGG enrichment analysis of all DEGs at different time points under low-temperature stress in R998. (**e**) Line graph of the cluster analysis of all DEGs at different time points under low-temperature stress in R998. The red numbers represent the numbers of DEGs and TFs in each cluster.

The differential expression analysis of R998 at different time points under low-temperature stress revealed that 613 DEGs were identified at 1 h compared with 0 h, of which 513 were upregulated and 100 were downregulated, including 32 unique DEGs (Figure 4a,b). A total of 2343 DEGs were identified at 3 h, of which 1557 were upregulated and 786 were downregulated, including 163 unique DEGs. A total of 3829 DEGs were identified at 6 h, of which 2331 were upregulated and 1518 were downregulated, including 221 unique DEGs. A total of 7252 DEGs were identified at 12 h, of which 3799 were upregulated and 3453 were downregulated, including 883 unique DEGs. A total of 8685 DEGs were identified at 24 h, of which 4434 were upregulated and 4251 were downregulated, including 2722 unique DEGs. To elucidate the functions of the 10,741 DEGs in R998, we analyzed the enriched GO terms and KEGG pathways. The significantly enriched GO terms were response to stress, hormone-mediated signaling pathway, cell cycle process, intracellular signal transduction, cellular glucan metabolic process, hydrogen peroxide metabolic process, regulation of ion transmembrane transport, photosynthetic electron transport chain, polysaccharide biosynthetic process, and photosynthesis biological process (Figure 4c). The significantly enriched KEGG pathways included plant hormone signal transduction, photosynthesis, glycerophospholipid metabolism, circadian rhythm, the MAPK signaling pathway, phenylpropanoid biosynthesis, carotenoid biosynthesis, galactose metabolism, alpha–linolenic acid metabolism, and starch and sucrose metabolism (Figure 4d). The functional changes in genes under low-temperature stress in R998 at different time points were investigated by clustering the 10,741 DEGs into six clusters using the k-means clustering algorithm, and the number of TFs in each cluster was determined (Figure 4e). Cluster 1 presented the highest expression level at 24 h, with 171 TFs among the 1582 DEGs. Cluster 2 presented the highest expression level at 0 h, with 518 TFs among the 5083 DEGs. Cluster 3 presented the highest expression level at 3 h and 6 h, with 169 TFs among the 742 DEGs. Cluster 4 presented the highest expression level at 12 h, with 187 TFs among the 1030 DEGs. Cluster 5 presented the lowest expression level at 24 h, with 161 TFs among the 1450 DEGs. Cluster 6 presented the lowest expression level at 6 h and 12 h, with 201 TFs among the 1030 DEGs.

### 2.3. Analysis of Differential Expression Between Cultivars

**Figure 5:**
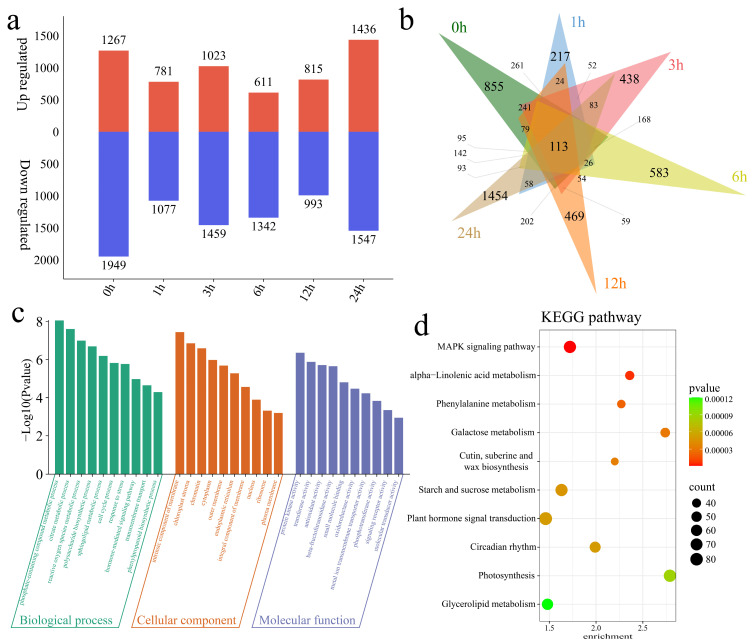
Analysis of differential expression and enrichment between GZW and R998. (**a**) Number of genes whose expression differed between GZW and R998. (**b**) Venn diagram of the numbers of common and specific DEGs between GZW and R998. (**c**) GO enrichment analysis of all DEGs between GZW and R998. (**d**) KEGG enrichment analysis of all DEGs between GZW and R998.

Analysis of the DEGs between GZW and R998 at the same time point of cryogenic treatment identified 3216 DEGs at 0 h, of which 1267 were upregulated and 1949 were downregulated, including 855 unique DEGs (Figure 5a,b). A total of 1858 DEGs were identified at 1 h, of which 781 were upregulated and 1077 were downregulated, including 217 unique DEGs. A total of 2482 DEGs were identified at 3 h, of which 1023 were upregulated and 1459 were downregulated, including 438 unique DEGs. A total of 1953 DEGs were identified at 6 h, of which 611 were upregulated and 1342 were downregulated, including 583 unique DEGs. A total of 1808 DEGs were identified at 12 h, of which 815 were upregulated and 993 were downregulated, including 469 unique DEGs. A total of 2983 DEGs were identified at 24 h, of which 1436 were upregulated and 1547 were downregulated, including 1454 unique DEGs. To elucidate the functions of the 7483 DEGs between the cultivars, we analyzed the enriched GO terms and KEGG pathways. The significantly enriched GO terms were phosphate-containing compound metabolic process, citrate metabolic process, reactive oxygen species metabolic process, polysaccharide biosynthetic process, sphingolipid metabolic process, cell cycle process, response to stress, hormone-mediated signaling pathway, transmembrane transport, and phenylpropanoid biosynthetic process (Figure 5c). The significantly enriched KEGG pathways were the MAPK signaling pathway; alpha-linolenic acid metabolism; phenylalanine metabolism; circadian rhythm; starch and sucrose metabolism; photosynthesis; plant hormone signal transduction; galactose metabolism; cutin, suberin, and wax biosynthesis; and glycerolipid metabolism (Figure 5d).

**Figure 6:**
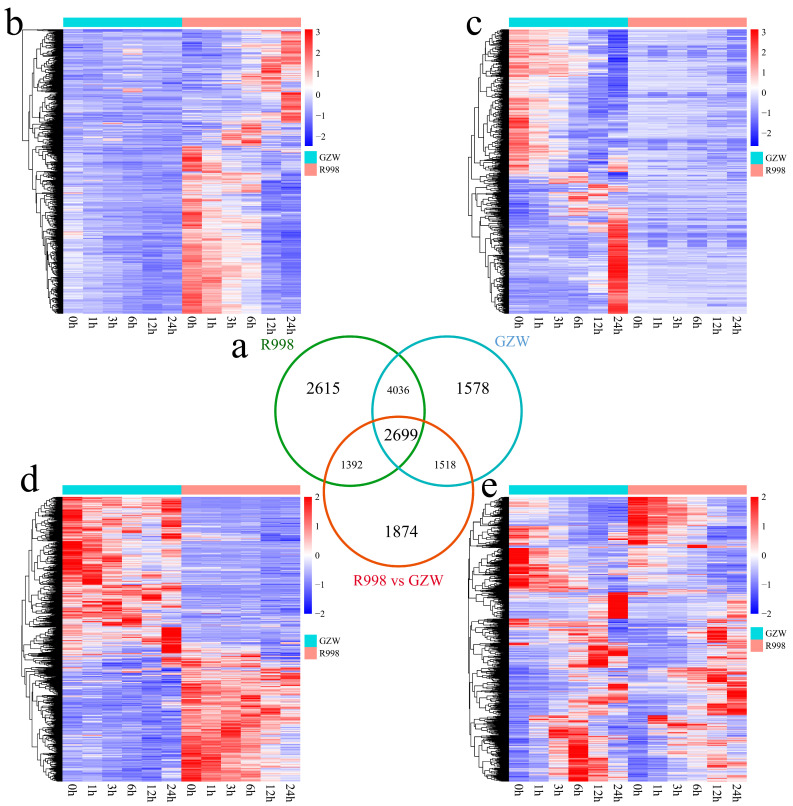
Heatmap of the numbers and expression patterns of common and unique DEGs between GZW and R998 and at different time points of low-temperature stress in the same material. (**a**) Venn diagram of common and specific DEGs between the GZW and R998 cultivars. (**b**) Heatmap of the expression patterns of DEGs uniquely expressed by R998. (**c**) Heatmap of the unique DEG expression patterns of GZW. (**d**) Heatmap of the unique DEG expression patterns between R998 and GZW. (**e**) Heatmap of DEG expression patterns common to R998 and GZW.

Furthermore, a total of 15712 DEGs were identified between and within cultivars, and 2615 unique DEGs were identified in R998, most of which were expressed at 0 h and exhibited a gradual decrease in expression with increasing duration of low-temperature stress (Figure 6). A total of 1578 unique DEGs were identified in GZW, approximately half of which presented the highest expression level at 0 h and only approximately half of which presented the highest expression level at 24 h with increasing duration of low-temperature stress. There were 1874 unique DEGs between R998 and GZW; approximately half of these DEGs were most highly expressed in R998, and approximately half were most highly expressed in GZW. A total of 2699 DEGs were differentially expressed not only between cultivars but also at different time points in the same material under low-temperature treatment.

### 2.4. Transcription Factors (TF) Analysis

**Figure 7:**
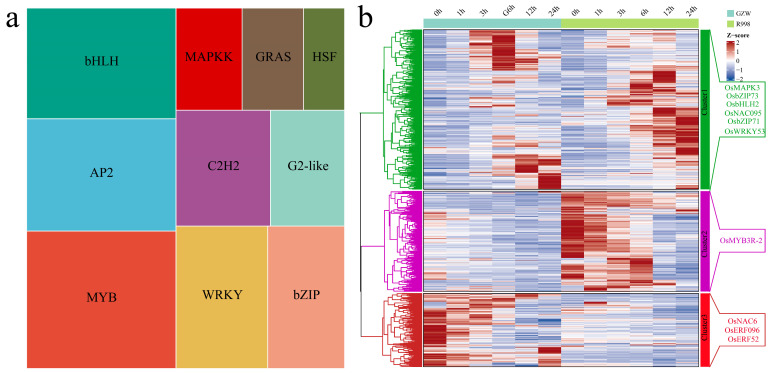
Differential expression of TFs and expression profile analysis. (**a**) Heatmap of the proportions of the top 10 TFs; the area represents the number of TFs, and different colors represent different TFs. (**b**) Clustering heatmap of TFs, with identified and validated TFs on the right.

A total of 15712 DEGs were identified during the same developmental period and development time point in the same material, including 1937 differentially expressed TFs. The 2696 differentially expressed TFs identified included bHLH, AP2, MYB, WRKY, bZIP, C2H2, G2-like, MAPKK, GRAS, and HSF TFs (Figure 7a). A total of three statistically significant clusters were identified from the 1937 differential TFs via the k-means clustering method (Figure 7b). The cluster 1 genes were differentially expressed between cultivars and at different cryogenic treatment periods in the same material, with the highest expression observed at 3 h, 6 h, 12 h, and 24 h in GZW and R998, and six TFs (*OsMAPK3, OsbZIP73, OsbHLH2, OsNAC095, OsbZIP71* and *OsWRKY53*) were identified and verified [17,18,19,20,21,22,23]. Cluster 2 presented the highest expression level in R998, with the expression gradually decreasing with low-temperature treatment, and a TF (*OsMYB3R-2*) was identified and verified [24]. Cluster 3 was the most highly expressed in GZW, and under low-temperature treatment, three TFs (*OsNAC6*, *OsERF096* and *OsERF52*) were identified and verified [25,26,27].

### 2.5. WGCNA

**Figure 8:**
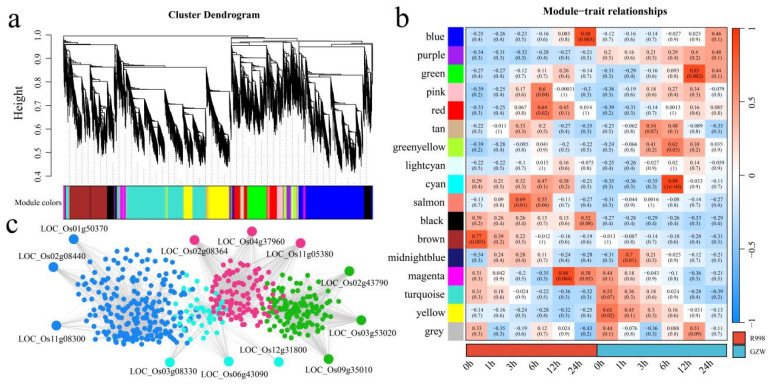
WGCNA and candidate gene mining. (**a**) Cluster dendrogram of all DEGs via WGCNA. (**b**) Correlation heatmap of the module with GZW and R998 at different time points of low-temperature stress. (**c**) Gene coexpression networks of significantly related modules.

To construct a gene coexpression network related to low-temperature tolerance in rice and mine the core genes from it, an expression matrix of 15,712 DEGs was used for the WGCNA, and the optimal soft threshold β = 15 was selected to construct a gene coexpression network related to low-temperature tolerance in rice via the dynamic tree cutting method. A total of 17 different coexpression modules (Figure 8a) were identified. According to the analysis of the correlations between the module and the two cultivars at different times, the blue module was significantly correlated with R998 at 24 h, the green module was significantly correlated with GZW at 12 h, the cyan module was significantly correlated with GZW at 12 h, and the magenta module was significantly correlated with R998 at 12 h (Figure 8b). In each module, the three genes with the highest degree of linkage were identified as candidate genes. The candidate genes identified in the blue module were *LOC_Os01g50370* (*OsMAPKKK63*), *LOC_Os02g08440* (*OsWRKY71*), and *LOC_Os11g08300* (*OsALDH3H2*) (Figure 8c). The candidate genes identified in the green module were *LOC_Os02g43790* (*OsERF91*), *LOC_Os03g53020* (*OsbHLH148*), and *LOC_Os09g35010* (*OsERF31*). The candidate genes identified in the cyan module were *LOC_Os03g08330* (*OsJAZ4*), *LOC_Os06g43090* (*OsMYB102*), and *LOC_Os12g31800* (*OsGRP6*). The candidate genes identified in the magenta module were *LOC_Os02g08364* (*OsPP2C11*), *LOC_Os04g37960* (*OsZEP1*), and *LOC_Os11g05380* (*OsCYP94C2*). Among these genes, *OsWRKY53* has been identified and verified in previous studies; this gene regulates the GA content in anthers by inhibiting the expression of GA biosynthetic genes, thereby negatively regulating the cold tolerance of rice at the booting stage [28].

### 2.6. qRT–PCR

**Figure 9:**
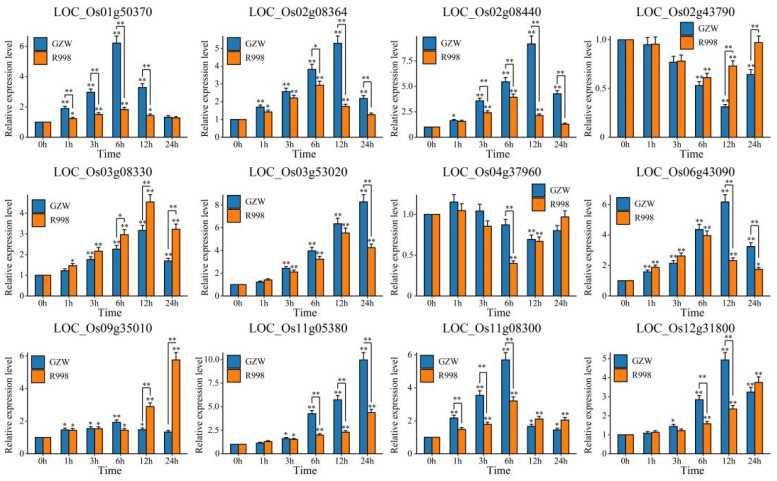
Analysis of the expression patterns of 12 candidate genes after low-temperature stress. The error bars represent the means of triplicates ± SEs (* *p* < 0.05, ** *p* < 0.01).

qRT–PCR was used to determine the expression patterns of these 12 candidate genes under low-temperature stress in the two cultivars (Figure 9). Compared with that at 0 h, the expression of 10 genes (*LOC_Os01g50370*, *LOC_Os02g08364*, *LOC_Os02g08440, LOC_Os03g08330*, *LOC_Os03g53020*, *LOC_Os06g43090, LOC_Os09g35010, LOC_Os11g05380, LOC_Os11g08300,* and *LOC_Os12G31800*) significantly increased under low-temperature stress (Figure 9). The expression of eight genes (*LOC_Os01g50370, LOC_Os02g08364, LOC_Os02g08440, LOC_Os03g53020, LOC_Os06g43090, LOC_Os11g05380, LOC_Os11g08300,* and *LOC_Os12g31800*) was significantly greater in GZW than in R998, and the expression of two genes (*LOC_Os03g08330* and *LOC_Os09g35010*) was significantly greater in R998 than in GZW. The expression levels of two genes (*LOC_Os02g43790* and *LOC_Os04g37960*) significantly decreased under low-temperature stress, the expression of *LOC_Os02g43790* in R998 was significantly greater than that in GZW, and the expression of *LOC_Os04g37960* in R998 was significantly lower than that in GZW.

**Figure 10:**
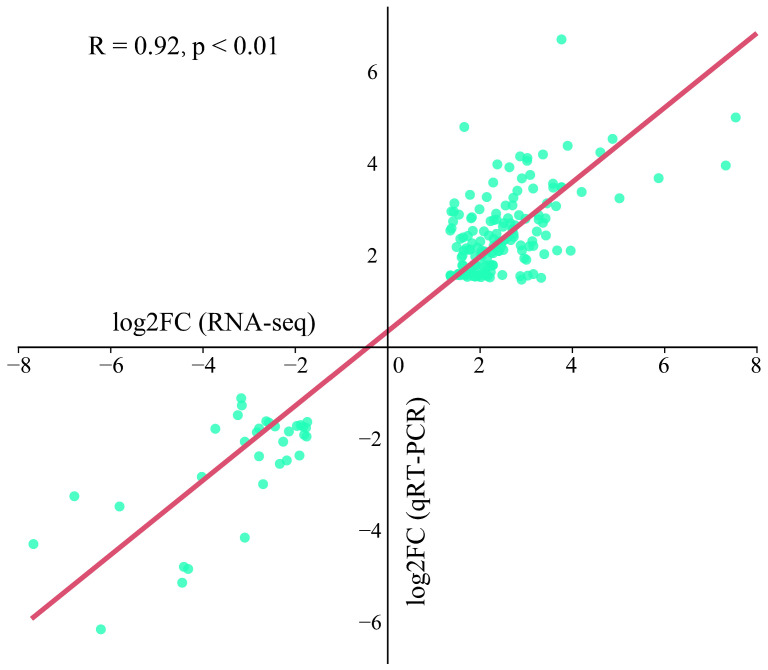
Scatter plot of the correlation between the qRT–PCR and RNA-seq data.

The fold correlation between the RNA-seq and qRT–PCR data for these 12 genes was further calculated, and the results revealed that the RNA-seq data were significantly correlated with the qRT–PCR data (R = 0.92, *p* < 0.01), which indicated that the transcriptome sequencing data were reliable (Figure 10).

## 3. Discussion

As a short-day crop, rice has stringent requirements for temperature adaptability, and in the rice production process, frequent “inverted spring cold” leads to rot in seeds and seedlings and even seedling death [2]. With global climate change, the early planting and double cropping of rice has generally increased, and even the seedling growth time in some areas has increased by more than a month, which further increases the possibility of rice plants experiencing low temperatures at the seedling stage. In addition, rice has long been domesticated and subjected to artificial selection, and the cultivation area has expanded [28]. Therefore, it is highly important to explore and analyze the molecular mechanism of rice cold tolerance to expand the planting area, ensure food security, and promote economic development. When rice plants are exposed to low-temperature stress, the water absorption capacity and transpiration both decrease, but the decrease in water absorption is greater than that in transpiration, which destroys the water metabolism balance in rice plants and leads to water loss in rice cells [29]. The effect of low-temperature stress on plant leaves directly reflects the ability of plants to cope with and resist low-temperature stress [30]. Rice is a short-day crop that is sensitive to temperature and humidity. Low temperatures affect the germination rate of rice, cause uneven germination, increase respiration, and increase endosperm consumption; therefore, low temperatures are not conducive to the growth of rice seedlings [3]. Leaves can directly reflect the degree of low-temperature injury and low-temperature stress resistance, and low-temperature stress can lead to changes in the apparent morphological characteristics of rice; however, the apparent morphology of different rice varieties in low-temperature environments is not consistent [31]. Changes in leaf morphology and damage to the cell structure caused by low-temperature stress may be related to the interactions of multiple physiological and biochemical mechanisms. For example, the curling and yellowing of leaves may be attributed to water imbalance and limited photosynthesis within leaf cells [15]. The weakening of transpiration induced by low temperatures leads to reduced water evaporation and accumulation of water inside the leaves, which may lead to leaf curling. Moreover, the inhibition of photosynthesis by low temperature reduces the chlorophyll content in leaves, resulting in yellowing of the leaves [32]. We found that there were differences in the apparent morphology and growth state between R998 and GZW under low-temperature stress. After 7 days of treatment at 10 °C, R998 resulted in obvious yellowing and greening of the leaves, while GZW resulted in high low-temperature resistance, and the leaves of GZW maintained their normal morphology and exhibited no yellowing. These results indicated that GZW had better adaptability and resistance to low-temperature stress than R998 and was better able to maintain its growth state. To analyze the transcriptome dynamics of rice with low-temperature tolerance, PCA and hierarchical clustering of samples of the two cultivars from the six time points revealed that the samples of GZW treated at low temperature for 6 h clustered far from those of R998 treated at low temperature for 6 h, and the samples of GZW treated at low temperature for 12 h clustered close to those of R998 treated at low temperature for 24 h, indicating that the main reason for the difference in low-temperature tolerance between R998 and GZW may be the difference between them after 6 h of low-temperature stress.

The study of pathways related to low-temperature tolerance in plants has long been a hot topic in the fields of plant biology and agricultural science. As one of the important technologies for mining genes and important analogs, RNA-seq has been used in the study of rice cold tolerance. On the basis of RNA-seq data from 11 indica and japonica rice materials under cold stress, a coexpression network of rice genes under cold stress was constructed, and the two genes, *OsCATC* and *Os03g0701200,* were confirmed as new candidate genes for improving rice cold tolerance [15]. Previous studies revealed that low temperature altered the expression of genes related to fatty acid, amino acid, carbohydrate, and energy metabolism in plants, and genes related to stock degradation, protein folding and stress response were also significantly affected, which was consistent with the results of this study [32,33]. The DEGs identified were significantly enriched in the phenylpropanoid biosynthetic process, regulation of ion transmembrane transport, response to stress, photosynthesis, glycerophospholipid metabolism, and starch and sucrose metabolism pathways. These findings indicate that under low-temperature conditions, the growth and metabolism of rice are affected, and photosynthesis can provide the energy required for rice, allowing it to maintain normal growth and metabolism. Under low-temperature stress conditions, the activity of enzymes required for photosynthesis reactions in plants is reduced, which affects the photosynthesis of plants, while low-temperature tolerant plants contain higher photosynthetic rates and can produce more sugars to provide energy to help them withstand the challenges of low temperature environments [34,35,36,37]. *OsPUS1* encodes a pseudouridine synthase located in the chloroplast, and its mutation affects chloroplast ribosome biogenesis, leading to the accumulation of superoxide anions in the body and leaf whitening under low temperatures [38]. Taken together, these results show that chloroplast fixation and energy storage and the mitochondria’s release of stored energy for cellular utilization play important roles in the cold response process of rice.

Previous studies have shown that exogenous abscisic acid (ABA) can improve plant resistance to low-temperature stress and that low-temperature stress can increase the ABA content in plants [39]. ABA can reduce water loss, inhibit plant growth, induce the expression of stress-related genes, and promote the adaptation of plants to stressful environments; thus, ABA is an important “antistress hormone” for plants. Under low-temperature stress, the level of ABA in rice also increases significantly [40]. At low temperatures, increasing the ABA content strengthened the ABA signal, and increasing the ABA content increased the expression of genes related to low-temperature tolerance in the downstream ABA response, thereby increasing the tolerance of rice to low-temperature stress. *OsbZIP73* was first identified via genome-wide association study (GWAS) analysis of indica-japonica differentiation genes that control cold tolerance at the seedling stage, and in-depth studies revealed that *OsbZIP73* can directly bind to the promoters of ABA biosynthetic genes (*NCED3* and *NCED5*), inhibiting ABA content to regulate rice cold tolerance [41]. *OsbZIP73* also regulated the expression of *qLTG3-1Nip*, a gene that regulates cold tolerance in rice seedlings, to affect sugar transport and distribution. *OsbZIP73* was also identified via RNA-seq, and we screened two ABA pathway genes (*OsPP2C11* and *OsZEP1*) as candidate genes for cold tolerance in rice. The expression of the ABA signal transduction gene *OsPP2C11* increased significantly with low-temperature stress [42]. These results indicated that under low-temperature stress, rice cold tolerance was improved by increased expression of genes in the ABA signal transduction pathway.

At present, it is generally believed that the response of plants to abiotic stress is regulated mainly by TFs. In studies of the effects of low temperatures on rice belts, many TFs have been identified and verified, and 10 of the 1937 TFs that we identified have been identified with functional validation in previous studies. The expression of *OsWRKY53* was upregulated by low-temperature induction at the booting stage, and the *wrky53* mutant presented a greater seed setting rate and greater pollen vigor than did the wild-type plants after low-temperature stress at the booting stage. Further studies revealed that *OsWRKY53* could directly bind to GA synthesis genes (*GA20ox1*, *GA20ox3,* and *GA3ox1*) and inhibit their transcriptional activity [21]. Genetic experiments also revealed that *OsWRKY53* is located upstream of the GA biosynthesis-related gene involved in the regulation of cold tolerance at the booting stage in rice. Therefore, *OsWRKY53* negatively regulates the cold tolerance of rice at the booting stage by inhibiting the expression of GA biosynthetic genes to regulate the content of GA in anthers [43]. The survival rate, photosynthetic capacity, fresh weight, and dry weight of the *OsWRKY71*-overexpressing plants were greater than those of the control plants after low-temperature (4 °C) treatment. *OsWRKY71* was also identified as a candidate gene for cold tolerance in rice [44]. *OsbHLH148* participates in the jasmonic acid pathway to improve the drought resistance of rice and is part of the *OsbHLH148-OsJAZ-OsCOI1* signaling module of rice [45]. Moreover, the candidate gene we identified, *OsMYB102*, was able to directly inhibit ABA accumulation via the transcriptional inhibition of the ABA catabolic enzyme *OsCYP707A6*. *OsMYB102* also indirectly inhibits ABA response genes, such as *OsABF4* and *OsNAP* [46]. As a gene coexpression network analysis method, WGCNA has many advantages but also has several disadvantages. When constructing a coexpression network, it is necessary to select corresponding parameters, such as appropriate thresholds and weight functions [46]. Different parameter selections may lead to different network structures, which in turn affect the subsequent analysis results. WGCNA decomposes the coexpression network into multiple modules, but the results of module division and the size of the modules are sensitive to the initial parameters, resulting in instability in module division. This means that different module division results may appear in different runs, which affects the subsequent functional annotation and interpretation. Too many modules may contain multiple unrelated genes, whereas too few modules may lead to the loss of key genes. Although WGCNA can reveal patterns and functions in gene coexpression networks, the specific explanations of these patterns and functions still require further in-depth analysis and biological experimental verification. Through WGCNA, we identified other candidate genes, such as *OsMAPKKK63, OsERF91, OsbHLH148, OsERF31,* and *OsMYB102*. The functions and mechanisms of action of these genes in the cold tolerance process of rice have not been verified. Only a few cold tolerance genes have been identified at the seedling stage. Therefore, it is highly practical to identify more cold tolerance genes and create new cold-tolerant rice germplasms. We identified several new genes that can be used as candidate genes for low-temperature tolerance in rice and subsequently carried out functional verification and molecular mechanism analysis of these important genes to aid in the molecular breeding of cold-tolerant rice.

## 4. Materials and Methods

### 4.1. Plant Material

In this study, plump seeds of indica rice (R998) and wild rice (GZW) were sown in a hydroponic box containing 96 wells after routine sterilization, soaking, and germination. A total of 480 seedlings of each variety were transferred to light incubators for treatment, and the temperatures in the incubators were set at two levels: a low temperature (constant temperature of 10 °C) and a suitable temperature (constant temperature of 25 °C, CK). The light intensity in the incubator was 31.2 (umols m^−2^s^−1^), with a 12 h/12 h light/dark cycle, and the relative humidity was 75%. Leaf samples were taken at 0 h, 1 h, 3 h, 6 h, 12 h, and 24 h after treatment, flash-frozen with liquid nitrogen, and stored in a −80 °C freezer for RNA extraction and physiological marker determination. The plants were incubated at 10 °C or 25 °C for 7 days, after which their growth was restored at 25 °C for 7 days.

### 4.2. Pro, POD, SOD, CAT and MDA Activities and Chlorophyll Content Determination

A total of 0.1 g of sample to be tested was added, 1 mL of extract was added, and ice bath homogenization was used for the subsequent determination of different enzyme activities. The mixture was centrifuged at 4 °C × 12,000 rpm for 10 min, the supernatant was removed, a water bath was used at 95 °C for 30 min, the mixture was cooled on ice, the mixture was centrifuged at 25 °C and 12,000 rpm for 10 min, 200 μL of the supernatant were collected, the absorbance at 532 nm and 600 nm was measured, and the activity of the MDA content was calculated [47]. The mixture was centrifuged at 4 °C × 12,000 rpm for 10 min, and the supernatant was taken as the liquid to be tested. The absorbance value A1 was immediately read at 470 nm, A2 was read after 1 min, and the POD activity was calculated [48]. After the supernatant was placed in a 37 °C water bath for 30 min, the absorbance value was measured at 450 nm to calculate the SOD activity [49]. After being extracted in a boiling water bath with shaking for 10 min at 25 °C × 12,000 rpm and centrifuged for 10 min, 10 μL of the supernatant and 190 μL of working solution were mixed, the initial absorbance value A1 at 240 nm and the absorbance value A2 after 1 min were measured, and the CAT activity was calculated [50]. Two hundred microliters of the supernatant was removed, the absorbance value was measured at 520 nm, and the Pro activity was calculated [47]. A 0.1 g sample was added to 1 mL of extraction buffer and placed in a −40 °C grinder under dark conditions for thorough grinding. The volume of the extract was rinsed into a 10 mL centrifuge tube. After extraction in the dark for 3 h (mixing 2 times during the period), 200 μL of extract and 200 μL of extraction buffer were added to the bottom residue, the mixture was recorded as a measurement tube and a blank tube, the absorbance values were read at 665 nm and 649 nm, respectively, and the chlorophyll content was calculated [51]. Each experiment was repeated three times.

### 4.3. RNA-Seq Library Preparation and Sequencing

The samples were transferred on dry ice to Beijing Group Biotechnology Co., Ltd. (Beijing, China) for RNA-seq sequencing. RNA extraction was performed via Invitrogen’s TRIzol (Thermo Fisher Scientific, Waltham, MA, USA) reagent extraction method. A certain amount of the total RNA extracted was fragmented. The disrupted mRNA was mixed well with primers, and first-strand cDNA was synthesized via PCR. Then, the two-chain synthesis reaction system was used to carry out two-chain reverse transcription, and two-chain product recovery was carried out. The cDNA obtained from reverse transcription was then end-modified, and A bases and adaptors were added. The ligation products were then amplified via PCR, purified, recovered, and labeled to complete cDNA library preparation. Library analysis was performed via an Agilent 2100 (Agilent Technology Co., Ltd., Santa Clara, CA, USA) and Q–PCR. The constructed libraries were sequenced on the Illumina HiSeq 2500 sequencing platform. After the original sequence was obtained, Fastp software (version 0.23.4) was used to remove adaptor sequences and filter out reads with low masses and those with high proportions of N sequences, obtaining clean reads that could be used for subsequent analysis [52]. HISAT2 was used to align the clean reads to the reference genome of rice [53]. We used featureCounts to compare and quantify the results [54].

### 4.4. Differential Expression Analysis

The unnormalized read count data were used as input data to calculate the *p* value and fold change values via DESeq2 software, and a *p* value < 0.05 and |log2fold change| > 1 were used as the threshold criteria to identify DEGs [55]. Enrichment analyses of the DEGs using the Gene Ontology (GO) and Kyoto Encyclopedia of Genes and Genomes (KEGG) databases were conducted via the clusterProfiler software (version 4.14.3) package in R language (version 4.4.2) [56].

### 4.5. WGCNA

The expression profiles of DEGs were determined via dynamic branching and coexpression analysis using the R language WGCNA package [57]. The criteria used were a weighting coefficient close to 0.8 and meeting of the correlation coefficient requirement; β = 14 was selected as the weighting coefficient in this study. Blockwise modules were used to construct a network to obtain gene coexpression modules (minModuleSize = 30 and Merge Cut Height = 0.25). The correlation coefficient and *p* value between the characteristic vector ME (Module Eigengene) of the module and Verticillium cultured at different temperatures were calculated. Visualization of the coexpression networks was performed via Cytoscape software (version 3.9.0) [58].

### 4.6. qRT–PCR

According to the cDNA sequence of the gene, primers were designed for the specific region at the 5′ or 3′ end of the gene sequence via Primer 5.0 software (Table S1). Total RNA was extracted via RNA-Easy Isolution Reagent (Novezan Biologics, Nanjing, China), and reverse transcription was performed using HiScript II Q Select RT SuperMix for qRT–PCR (+gDNA wiper) (Novozan Biologics). A real-time PCR (qRT–PCR) assay was performed with Mona Biologics MonAmp™ SYBR^®^ Green qRT–PCR Mix (Monad Biotech Co., Ltd., Suzhou, China) using cDNA as a template, with a total system volume of 10 μL. The reaction procedure was as follows: predenaturation at 95 °C for 30 s, 40 cycles of denaturation at 95 °C for 10 s, annealing at 60 °C for 30 s, and extension at 72 °C for 20 s. The 2^−ΔΔCt^ method was used for relative quantitation, and the internal reference gene was *Ubiquitin* (*LOC_Os03g13170*) [59].

## 5. Conclusions

In conclusion, the phenotypic evaluation of low-temperature stress in indica rice (R998) and wild rice (GZW) and the RNA-seq data at six time points provide a reliable dataset for the study of cold tolerance in rice. By identifying DEGs and TFs, phenylalanine metabolism, photosynthesis, plant hormone signal transduction, and starch and sucrose metabolism pathways were closely related to low-temperature tolerance in rice. In addition, we constructed a gene regulatory network for low-temperature tolerance in rice via WGCNA and identified 12 key genes for low-temperature tolerance, including five TFs, one of which was identified and verified in previous studies. However, the exact role of these genes in the tolerance of rice to low temperatures remains to be determined. These results provide a theoretical basis for an in-depth understanding of the molecular mechanism of low-temperature tolerance in rice and provide new genetic resources for the study of low-temperature tolerance in rice.

## Data Availability

The RNA-seq data used in the study have been uploaded to the China National Center for Bioinformation database (https://ngdc.cncb.ac.cn/gsa/s/v6uG47wT, accessed on 18 November 2024) with the accession number CRA019091.

## Supplementary Materials

The following supporting information can be downloaded at: https://www.mdpi.com/article/10.3390/ijms252413380/s1.
