# Supplementary figures and images for: Comparative Transcriptomic Analysis and Candidate Gene Identification for Wild Rice (GZW) and Cultivated Rice (R998) Under Low-Temperature Stress
Source: Int J Mol Sci. 2024 Dec 13;25(24):13380. doi: 10.3390/ijms252413380 (PMC11676510; doi:10.3390/ijms252413380)

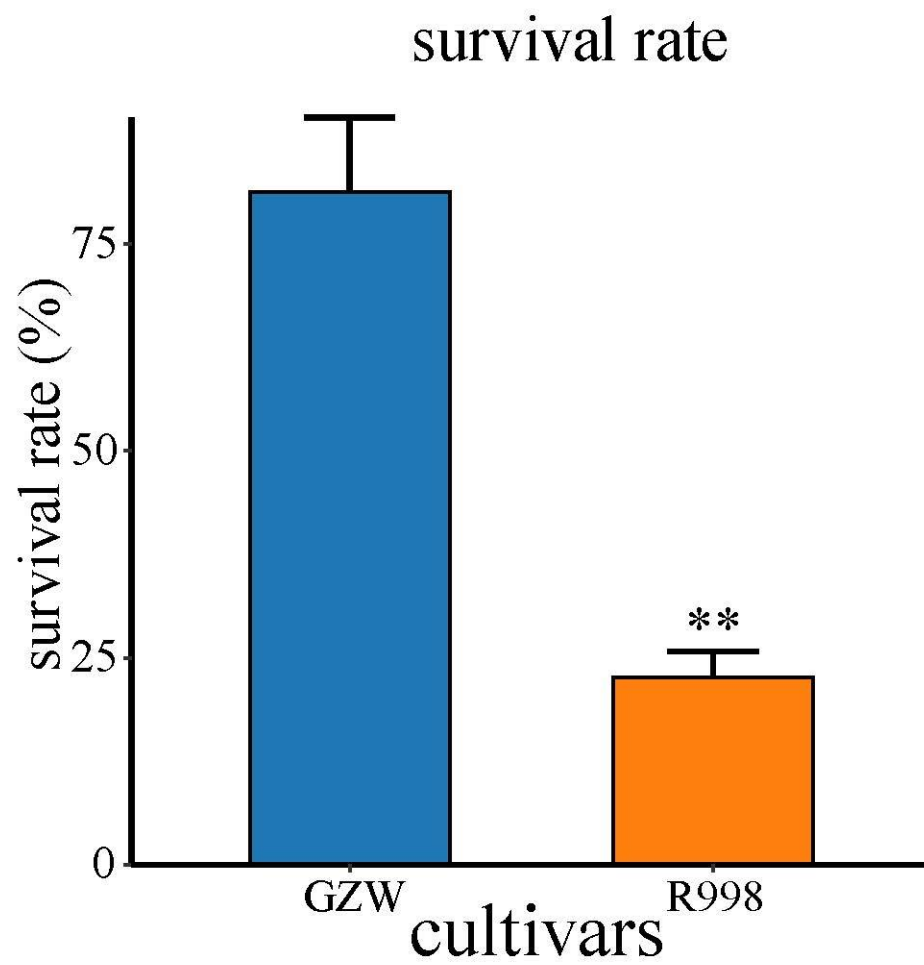

**Figure S1.** R998 and GZW survival rate after 7 days of 10 C treatment and 25 C treatment for 7 days.

Supplement: Supplementary file 1 [file ijms-25-13380-s001.zip › Figure S1.pdf]
